# Network Structure of Nomophobia, Fear of Missing Out, Mindful Attention, and Happiness Among University Students

**DOI:** 10.3390/bs16091613

**Published:** 2026-09-09

**Authors:** Mahmood Salim Almaawali, Gomaa Said Mohamed Abdelhamid, Muna Al-Bahrani, Yousef Abu Shindi, Yusen Zhai, Manal Al-Fazari, Suhail Al-Zoubi

**Affiliations:** 1 Department of Psychology, College of Education, Sultan Qaboos University, P.O. Box 32, Muscat 123, Oman; munabh@squ.edu.om (M.A.-B.); yousefaaa@squ.edu.om (Y.A.S.); manal@squ.edu.om (M.A.-F.); smalzoubi@squ.edu.om (S.A.-Z.); 2 Educational Studies Department, Rustaq College of Education, University of Technology and Applied Sciences, P.O. Box 10, Rustaq 329, Oman; 3 Department of Educational Psychology, Faculty of Education, Fayoum University, Fayoum 63514, Egypt; 4 School of Human Development and Organizational Studies in Education, University of Florida, Gainesville, FL 32611-7042, USA; yusenzhai@ufl.edu

**Keywords:** nomophobia, fear of missing out, mindful attention, happiness, network analysis, gender differences

## Abstract

Nomophobia, fear of missing out (FoMO), mindful attention, and happiness are interconnected psychological constructs, yet their interdependencies remain poorly understood, particularly in Arab-speaking contexts. Using network analysis (EBICglasso), this cross-sectional study examined these associations in 462 Omani college students across a 56-node, four-community network. Centrality indices (strength, expected influence) and bridge expected influence were calculated, with stability assessed via bootstrapping and gender differences examined using the Network Comparison Test. Results revealed strong within-community connections and notable cross-community links. Mindful attention showed positive associations with nomophobia and FoMO but negative associations with happiness. Happiness and mindful attention emerged as the most central constructs, while FoMO and mindful attention showed the strongest bridge associations across network communities. Stability coefficients exceeded the recommended 0.50 threshold, and no significant gender differences emerged in network structure or global strength. These findings highlight the complexity of the associations among mindful attention, nomophobia, FoMO, and happiness and suggest that the role of mindful attention may vary across contexts. The identified central and bridge constructs warrant further investigation as potential areas of focus in future digital well-being research.

## 1. Introduction

Smartphone-related concerns such as nomophobia and fear of missing out (FoMO) have received increasing attention in relation to young adults’ mental health, particularly in the post-COVID era (Solano et al., 2021). Increased reliance on smartphones for social networking sites (SNSs) has intensified daily screen engagement (Zhang, 2023). Although SNS use may enhance perceived happiness through social connectedness (Akinci & Durmuş, 2025), much of the literature consistently links excessive smartphone use to poorer mental health outcomes (Al-Mamun et al., 2025; Alhusseini et al., 2025; Alhaj et al., 2024; Notara et al., 2021; Qutishat et al., 2020; Abel et al., 2016; Przybylski et al., 2013). A meta-analysis reported a global pooled prevalence of 26.99% for smartphone addiction, which has exceeded rates of social media, cybersex, and gaming addictions (Meng et al., 2022).

Nomophobia refers to anxiety arising from being unable to access one’s mobile phone (Yildirim & Correia, 2015), and FoMO reflects persistent concerns about missing rewarding social experiences, often amplified through social media (Przybylski et al., 2013). Both constructs are consistently associated with poorer psychological outcomes among young adults across cultural contexts (Notara et al., 2021; Al-Mamun et al., 2025; Qutishat et al., 2020; Alhaj et al., 2024; Alhusseini et al., 2025; Arampatzi et al., 2018; Kaya et al., 2020).

Prevalence estimates vary widely, with some systematic reviews reporting that more than 15−90% of young adults suffered from signs of nomophobia, and generally negative mental health outcomes were reported (Notara et al., 2021). In some cultures, nomophobia was reported in 94% of young adults (*n* = 36,656), half of whom had moderate nomophobia (Al-Mamun et al., 2025). In Oman, more than 90% of a surveyed sample of university students (*n* = 735) showed signs of nomophobia, 65% of which reported moderate levels (Qutishat et al., 2020). Students with severe nomophobia had reportedly lower GPA than higher achieving students. In the United Arab Emirates, about half (47.7%) of a university student sample (*n* = 232) had moderate levels of nomophobia, and higher stress and anxiety were reported as nomophobia increased (Alhaj et al., 2024). In Saudi Arabia, mixed findings were reported. Adults older than 50 had higher levels of nomophobia than young adults (age 18–29), and the higher the score was on NMPQ, the higher reported distress was, especially among male participants (mean score = 78.8) (Alhusseini et al., 2025).

Overall, although prevalence estimates vary considerably across populations and settings, existing evidence suggests that nomophobia is frequently associated with poorer mental health outcomes. It is clear, then, that nomophobia is generally associated with poor mental health in young populations, calling for a deeper understanding of nomophobia and its association with positive health indicators such as mindfulness and happiness. Evidence also suggests these associations may depend on contextual factors such as social connectedness or level of use (Arampatzi et al., 2018; Ren et al., 2026), pointing to a more conditional relationship than early formulations assumed. Indeed, recent work has proposed the concept of “functional nomophobia,” suggesting that moderate nomophobia may serve adaptive or socially integrative functions and become detrimental only when excessive or problematic (Ren et al., 2026). Taken together, these findings indicate that nomophobia is generally associated with poorer mental health in young populations, calling for a deeper understanding of its association with positive health indicators such as mindfulness and happiness.

FoMO describes the desire to remain continuously connected to online social life (Przybylski et al., 2013). FoMO is particularly relevant to young adults because social connection and continuous access to online social networks are central aspects of contemporary communication. Despite existing evidence that young people are most susceptible to FoMO, little attention has been directed to understand its relation to positive human experience. Much of the existing evidence suggests negative outcomes and emotional experiences. However, research has yet to study whether symptoms of FoMO (often believed to be negative) can contribute to positive emotions such as happiness. Being constantly in touch with social networks, even during holidays, might increase feelings of belongingness and inclusiveness. For young adults, a mobile phone may be more than just a device; it is their main tool for communication. Its role has increasingly become paramount for social interaction, and thus constant check of updates on social media may not indicate addictive behaviors (Arampatzi et al., 2018). Rather, continuous online social connection could buffer against anxiety and isolation, as social networks have consistently been linked to academic success and decreased mental health problems (Kaya et al., 2020).

In this context, mindful attention has been proposed as a potential protective factor relevant to the psychological experiences associated with nomophobia and FoMO. Mindfulness is defined as nonjudgmental awareness of the present moment and has been widely proposed as a protective factor for mental health (Kabat-Zinn, 2015). Higher dispositional mindfulness is associated with improved well-being (Karl et al., 2022), lower problematic smartphone use, and reduced boredom proneness (Regan et al., 2020). Mindful individuals may regulate smartphone use more intentionally rather than impulsively (Hefner & Freytag, 2024), and mindfulness-based approaches have been suggested to mitigate problematic smartphone use symptoms (Liu et al., 2022). In collectivistic cultures, mindfulness strongly predicted happiness (59%) and well-being (48%) regardless of gender, likely through attentional focus on the present moment and reduced stress reactivity (Aldahadha, 2023).

However, evidence concerning the relationship between mindfulness and technology-related constructs is not entirely consistent. Most existing research supports mindfulness as a protective factor against FoMO: mindful attitudes have been found to reduce FoMO both directly and through their association with secure attachment (Perazzini et al., 2023), and mindful attention has been shown to mediate the link between FoMO and passive social media use, with higher mindfulness predicting lower FoMO (Weaver & Swank, 2021). Similarly, meta-analytic evidence indicates mindfulness is associated with reduced FoMO and problematic technology use more broadly (Fendel et al., 2025). Nevertheless, some studies suggest this protective relationship is not uniform: mindfulness’s association with nomophobia, for instance, has been found to be significant only for women (Arpaci et al., 2017), suggesting the buffering effect of mindfulness may be conditional on individual or contextual factors rather than universal. Taken together, these findings suggest that mindfulness may have complex, and not uniformly protective, associations with technology-related concerns and well-being—setting up the present study’s novel network-level finding of a positive mindfulness–FoMO association as a genuine departure from prior work rather than a replication of it.

Happiness is an important indicator of subjective well-being and has been examined as a positive dimension of psychological health (Genecov et al., 2024). Post-COVID-19, research started to explore whether young adults are happier with or without their mobile phones (i.e., Karagöz et al., 2025), and whether sources of happiness have changed over time (Genecov et al., 2024). In some populations, young adults tend to report high happiness even if they report high problematic mobile phone use (Kaya et al., 2020). Therefore, the relationship between smartphone-related concerns and happiness may be more nuanced than a simple assumption that greater smartphone-related problems necessarily correspond to lower happiness. Therefore, it is important to examine whether happiness is systematically associated with technology-related concerns such as nomophobia and FoMO.

Understanding the relationships among mindful attention, nomophobia, FoMO, and happiness is complex, given the interconnected nature of these constructs. A substantial body of research indicates that FoMO and nomophobia are closely interrelated and consistently associated with adverse mental health outcomes, including heightened anxiety, psychological distress, and reduced well-being or happiness (Ceobanu et al., 2023; Dalvi-Esfahani et al., 2019; Gaber Hamzaa et al., 2024; Holte, 2023; Karami et al., 2025; Musetti et al., 2022; Taylor-Jackson & Moustafa, 2021; Wan et al., 2024). These findings suggest that both constructs may function as cognitive–affective vulnerabilities linked to diminished psychological well-being.

However, emerging evidence complicates this largely negative narrative. A large-sample German longitudinal study found that SNS use did not generally predict happiness and was only associated with reduced happiness among individuals who were socially disconnected (Arampatzi et al., 2018). Similarly, recent research proposes the concept of “functional nomophobia,” suggesting that moderate levels of nomophobia may serve adaptive or socially integrative functions and become detrimental only when excessive or problematic (Ren et al., 2026). These findings imply that the relationship between technology-related anxieties and well-being may be contingent on contextual and individual factors.

Mindfulness adds another layer of complexity to this system. Research generally supports a positive association between mindful attention and happiness, with mindfulness potentially enhancing well-being through present-moment awareness and emotional regulation (Salvi et al., 2021). Moreover, mindfulness has been conceptualized as both a mediator and a moderator in the relationships among FoMO, nomophobia, and happiness, indicating that interventions aimed at increasing mindfulness may mitigate the negative effects of FoMO and nomophobia while promoting well-being (Duprey et al., 2018; Regan et al., 2020; Chang et al., 2023). Taken together, these mixed findings suggest the need for analytic approaches capable of modeling complex, bidirectional, and potentially conditional relationships among these constructs.

Taken together, these findings suggest that nomophobia, FoMO, mindfulness, and happiness may be interconnected in complex ways that are not adequately captured by examining isolated associations between total scores. Network analysis provides a complementary framework for examining the interconnected structure of these constructs and identifying central and bridge nodes across psychological domains (Abdelhamid et al., 2024).

### The Present Study

College students face unique challenges to maintain psychological well-being while achieving academic success. Excessive social media use may amplify these pressures through persistent peer comparison and poorer mental health (Abdoli et al., 2023). In collectivistic cultures where social connection is highly valued (Demir & Sümer, 2018), FoMO and nomophobia may relate to well-being in culturally specific ways, and mindful attention, as conceptualized by Kabat-Zinn, may be interpreted differently in Arab-speaking contexts. Nevertheless, research on mindfulness and happiness in Arab-speaking samples, particularly in relation to FoMO and nomophobia, remains scarce. Existing studies report limited effects of mindful attention on mental health (Al-Ghalib & Salim, 2018) and nonsignificant links between mindfulness and academic performance despite moderate mindfulness levels (Alomari, 2023).

To our knowledge, the present study is among the first to examine the associations among nomophobia, FoMO, mindful attention, and happiness at the item and construct levels using network analysis. Our goals are fourfold. First, we aim to explore the complex associations between nomophobia and FoMO as technology-related constructs and the well-being constructs (mindfulness and happiness), including whether these concerns may coexist with positive indicators of mental well-being. Second, we seek to identify central and bridging nodes within the network. Traditional approaches such as regression or structural equation modeling treat constructs as unified latent variables and may overlook nuanced item-level interactions (Borgatti et al., 2009). Network analysis allows for examination of the interconnected relationships among individual components of these constructs (Ozimek et al., 2025). Third, we assess network stability by evaluating the robustness of edge weights and centrality indices using resampling techniques. Fourth, we examine potential gender differences in network structure and global strength, exploring whether the associations among nomophobia, FoMO, mindfulness, and happiness differ between male and female students. By identifying central and influential nodes, this study aims to enhance understanding of the interrelationships among these four constructs and generate hypotheses for future research on digital well-being.

Based on the reviewed literature, we formulated the following research questions:

**RQ1.**  *What is the network structure of nomophobia, FoMO, mindfulness, and personal happiness at the item level, and which nodes are most central?*

**RQ2.**  *Which nodes function as cross-community bridges between technology-related constructs (nomophobia and FoMO) and well-being constructs (mindfulness and happiness)?*

**RQ3.**  *Does the network structure or global strength differ between male and female students?*

Given the mixed evidence on mindfulness and well-being in collectivistic cultures, we did not posit directional hypotheses for all associations but anticipated complex patterns of association among nomophobia, FoMO, mindfulness, and happiness, consistent with previous theoretical and empirical work on mindfulness (Britton, 2019).

## 2. Materials and Methods

### 2.1. Participants

A total of 488 students (63.9% female) participated in the study from a large public university in Oman. Students came from various colleges and represented a range of demographic backgrounds (see Table 1). All participants completed measures of mindfulness, nomophobia, FoMO, and happiness. No cases were removed due to missing data, and all participants provided informed consent prior to data collection. The study was approved by the Internal Review Board code (REAAF/EDU/PSYCHOLOGY/2024/07) before data collection.

### 2.2. Measures

#### 2.2.1. Nomophobia Questionnaire (NMP-Q)

Nomophobia refers to the discomfort or anxiety experienced when an individual is separated from, or unable to use, their mobile phone (Yildirim & Correia, 2015). The Nomophobia Questionnaire (NMP-Q) was originally developed by Yildirim and Correia (2015) to measure the level of attachment to mobile phone use. The NMP-Q is a widely used measure that has been validated in several contexts and translated into several languages. In the current study, we used the Arabic version of the NMP-Q, developed and validated by Al-Barashdi and Aldhafri (2020). In their Arabic adaptation, Items 13 and 18 were excluded, resulting in an 18-item measure (Al-Barashdi & Aldhafri, 2020). Using a large sample of college students (2507, of which 868 were males), the Arabic NMP-Q (18 items) demonstrated a good reliability (α= 0.87) (Al-Barashdi & Aldhafri, 2020). Furthermore, the Arabic NMP-Q revealed excellent internal consistency in the current study (α = 0.926 and McDonald’s ω = 0.925).

#### 2.2.2. Fear of Missing Out Scale (FoMO)

FoMO was measured using the 10-item FoMO scale (Przybylski et al., 2013). Individuals responded to statements reflecting FoMO-related cognitions, such as being afraid that their friends have more fun without them or fear of not being up to date with what is happening around them. The FoMO scale consisted of 10 items measured on a five-point Likert scale (e.g., 1 = not at all true of me to 5 = extremely true of me). Higher scores indicate greater FoMO (Przybylski et al., 2013). In the original version, internal consistency was strong (α = 0.87). The Arabic FoMO scale showed good internal consistency in the current study (Cronbach’s α = 0.781 and McDonald’s ω = 0.760).

#### 2.2.3. Mindful Attention Awareness Scale (MAAS)

The Mindful Attention Awareness Scale (MAAS) is a 15-item self-report measure assessing dispositional mindfulness, defined as open or receptive awareness of and attention to present-moment experiences in everyday life. Notably, the MAAS primarily assesses mindful attention and present-moment awareness rather than broader dimensions such as acceptance and non-judgment. Items are rated on a 6-point Likert-type scale ranging from 1 (Almost always) to 6 (Almost never), with higher scores indicating greater mindful attention and awareness. All 15 MAAS items are worded in a single direction, describing everyday lapses in attention (i.e., mindlessness); consequently, no items required reverse-scoring, as the response anchors establish the scoring direction. The composite MAAS score was calculated as the sum of all 15 item responses, yielding a possible total score range of 15–90, with higher total scores indicating greater dispositional mindful attention (Brown & Ryan, 2003). The MAAS primarily captures the attention–awareness component of mindfulness, making it suitable for examining relationships between mindfulness and other psychological constructs in network analysis. The scale has demonstrated strong internal consistency and construct validity across diverse samples (Brown & Ryan, 2003). The MAAS was translated into Arabic. Forward and backward translation was carried out by four psychologists. Post initial translation, the four professionals met to discuss similarities and discrepancies in translation in light of accepted forms of expression consistent with the local use of Arabic. Any items that were translated differently were retranslated during the session. Phase two of translation included sharing the translated version to another two experienced psychologists who were also provided with the original English items. Their feedback was incorporated into the final version.

**Figure 1:**
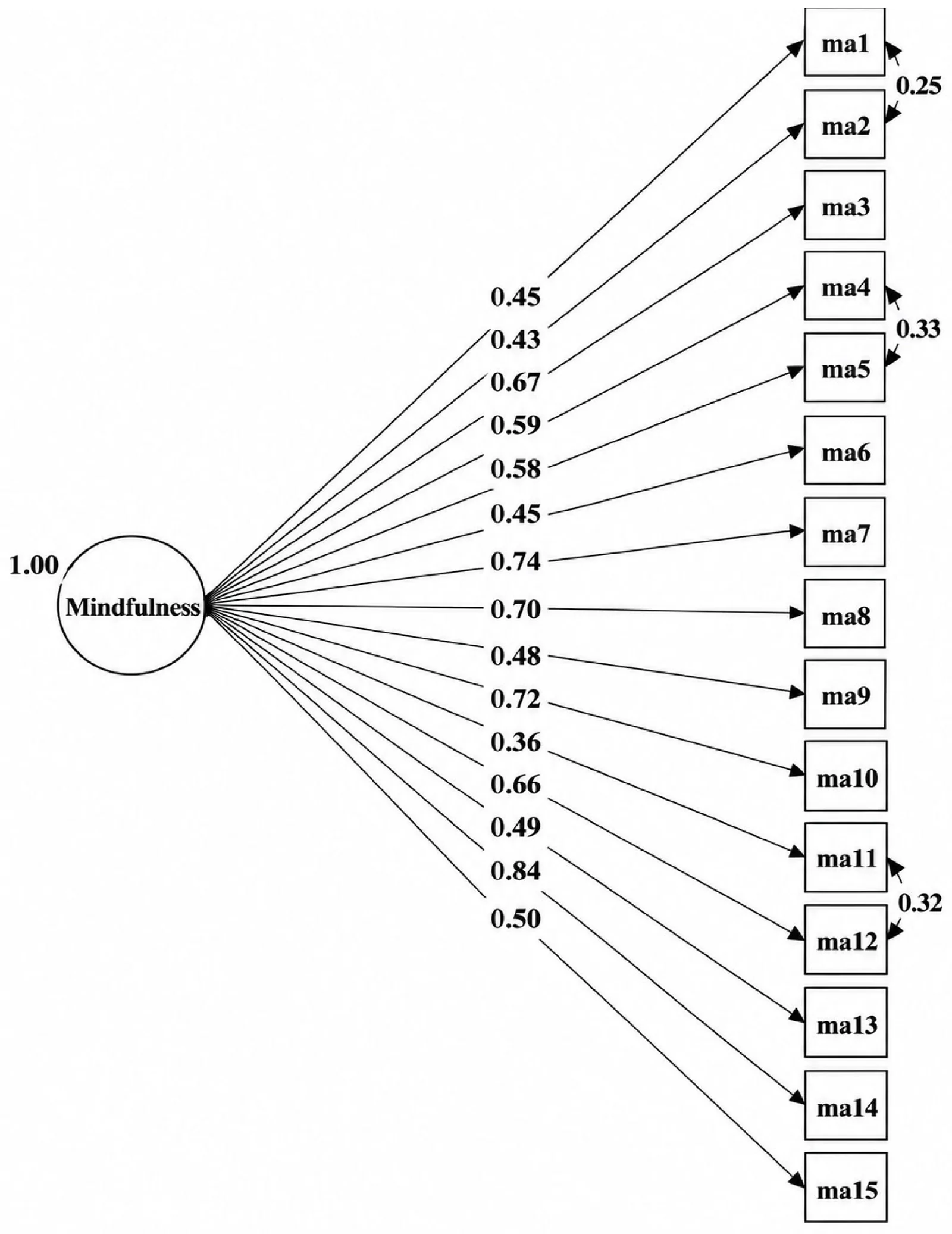
Confirmatory factor analysis model of the Mindful Attention Awareness Scale (MAAS).

In the current study, the factor structure of the Mindful Attention Awareness Scale (MAAS) was evaluated using confirmatory factor analysis (CFA) with the weighted least squares mean and variance adjusted (WLSMV) estimator in Mplus Version 8.3, using a randomly selected subsample of 286 students from the original dataset. A single-factor model was specified, with all 15 items loading on a latent mindfulness factor, with standardized factor loadings ranging from 0.36 to 0.84 (see Figure 1). 

The one-factor model demonstrated an acceptable fit to the data, χ^2^(87) = 253.39, *p* < 0.001, CFI = 0.946, TLI = 0.934, RMSEA = 0.082 (90% CI [0.070, 0.094]), and SRMR = 0.047. Internal consistency was assessed using both Cronbach’s alpha (α) and McDonald’s omega (ω), as recommended for providing complementary evidence of scale reliability (Abdelhamid et al., 2026). The translated MAAS demonstrated good internal consistency, with Cronbach’s α = 0.863 and McDonald’s ω = 0.862, supporting the reliability of the measure for use in the current sample.

#### 2.2.4. Personal Happiness Index (PHI-13)

Adapted from the Oxford Happiness Questionnaire (OHQ-29) (Hills & Argyle, 2002), the Personal Happiness Index (PHI-13) (Moeinaddini et al., 2020) was used to measure levels of perceived personal happiness among the sample. Using a sample of 300 Malay adults, the PHI-13 carried Structural Equation Modeling (SEM) on the OHQ-29 to assess the individual contribution of each item within the OHQ-29 in explaining personal happiness and to develop a new brief measure for personal happiness (Moeinaddini et al., 2020). Results showed that almost half (16) of the OHQ-29 items had weak contributions in the assessment of personal happiness. Items 3, 4, 8, 9, 11, 12, 15, 16, 17, 18, 21, 22, & 25 remained and now constitute the new PHI-13 (Moeinaddini et al., 2020). Although the alpha Cronbach’s of the new PHI-13 was not reported independently, the full set of items (29) in that sample was strong (α = 0.863), indicating strong reliability.

**Figure 2:**
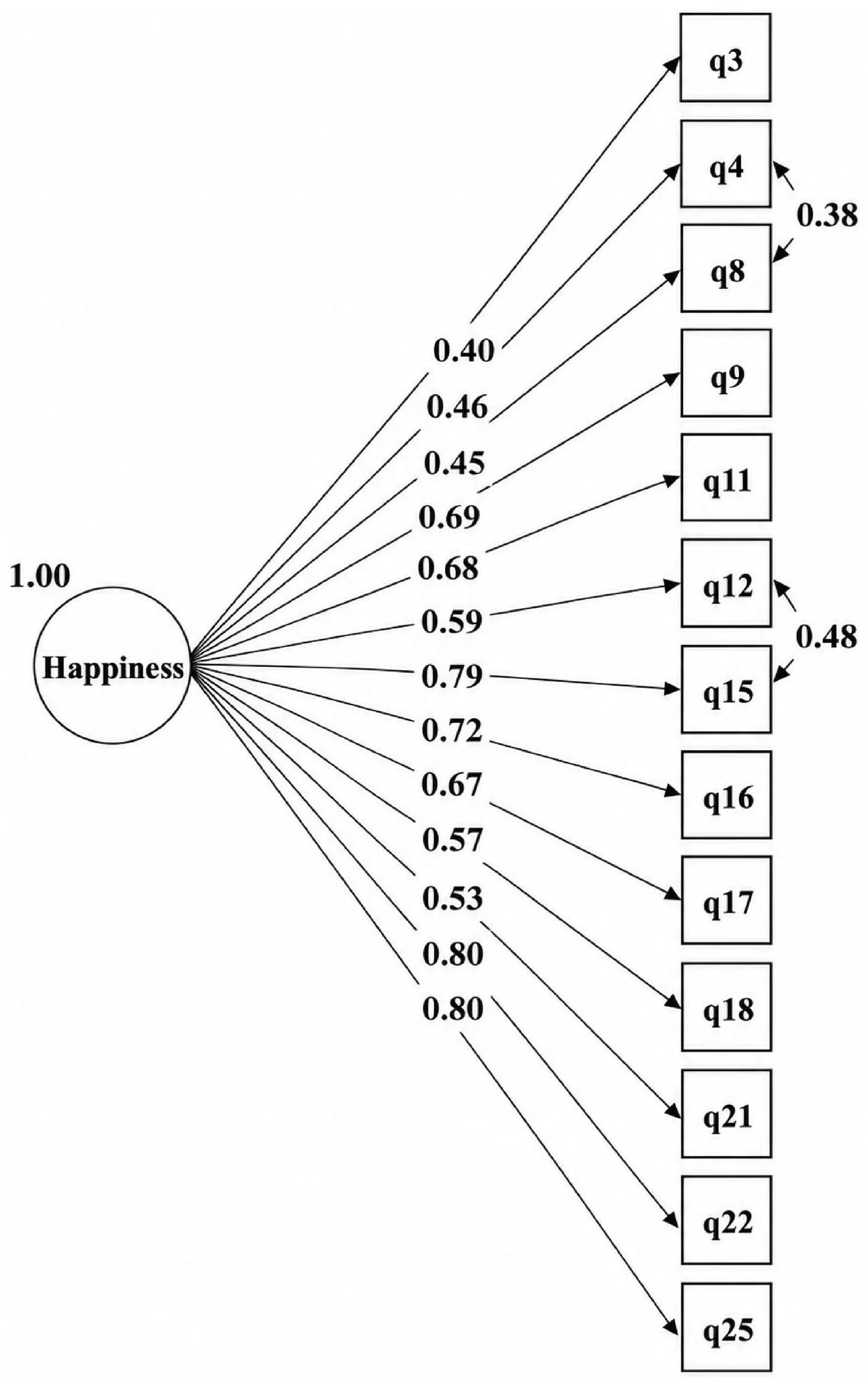
Confirmatory factor analysis model of personal happiness based on the PHI-13 items.

The factor structure of the Arabic PHI-13 was evaluated using confirmatory factor analysis (CFA) with the weighted least squares mean and variance adjusted (WLSMV) estimator in Mplus Version 8.3, using a randomly selected subsample of 286 students from the original dataset. All 13 items were specified to load on a single latent personal happiness factor, with standardized factor loadings ranging from 0.40 to 0.80 (see Figure 2). The one-factor model demonstrated an acceptable fit to the data, χ^2^(63) = 178.66, *p* < 0.001, CFI = 0.967, TLI = 0.960, RMSEA = 0.080 (90% CI [0.066, 0.094]), and SRMR = 0.040. The Arabic PHI-13 demonstrated good internal consistency in the current sample, with Cronbach’s α = 0.872 and McDonald’s ω 0.872.

### 2.3. Statistical Analysis

#### 2.3.1. Network Estimation

Network analysis provides a framework for examining patterns of associations among psychological variables, allowing the identification of highly connected or central variables within a network (Epskamp et al., 2018; Nuño et al., 2022). The network structure was estimated using a regularized partial correlation network model implemented through the Extended Bayesian Information Criterion graphical lasso (EBICglasso) from the qgraph package (1.9.8) in R (version 4.6.0) and RStudio (version 2025.09.2+418). The network consists of nodes and edges, where each node represents an item and the edges denote the associations among nodes. Green edges represent positive associations, while red edges represent negative associations. The network comprises a total of 56 nodes (items): 18 items from Nomophobia, 15 items from Mindfulness, 10 items from FoMO, and 13 items from Happiness. A tuning parameter (γ) was set to 0.5 to maintain an optimal balance between model complexity and simplicity, allowing clearer detection of the true interrelationships within the network (Epskamp et al., 2018). As a robustness check, a supplementary network analysis was conducted using polychoric correlations for ordinal variables, to account for the ordinal nature of the 5-point and 6-point Likert-type items. The results of this supplementary analysis are presented in the Supplementary Materials.

#### 2.3.2. Network Inference

To assess the importance of individual nodes within the network, we used the R packages qgraph (version 1.9.8) and networktools (version 1.6.0) to estimate Strength (ST), Expected Influence (EI), and Bridge Expected Influence (BEI) (Jones et al., 2021; van Borkulo et al., 2023). Strength reflects a node’s overall connectivity by summing the absolute values of its edge weights, while EI accounts for the direction of edges, summing the actual weights to capture both positive and negative associations (Opsahl et al., 2010; Epskamp et al., 2018). BEI quantifies a node’s role in linking distinct communities, reflecting both direct (1-step) and indirect (2-step) effects, and identifies nodes that serve as key connectors across traits or constructs (Jones et al., 2021). Nodes with higher BEI values exert greater influence and may facilitate the activation or maintenance of other traits. In this study, Mindfulness, Nomophobia, Fear of Missing Out, and Happiness were defined as four separate communities, with the top 20% of BEI values indicating key bridging nodes.

#### 2.3.3. Network Accuracy and Stability Estimation

The accuracy and stability of the estimated network were investigated using the R package bootnet (version 1.8) (Epskamp et al., 2018). Bootstrapped difference tests were carried out to evaluate the precision of edge weights, and 95% confidence intervals (CIs) were generated for each edge using 1000 bootstrap samples. Narrower CIs indicate more reliable edge estimates. To assess the stability of centrality indices, a case-dropping bootstrap method was employed to determine whether node centrality remains consistent when the network is re-estimated with progressively fewer observations.

Additionally, the correlation stability (CS) coefficient was calculated using the same case-dropping bootstrap approach with 1000 samples, providing a quantitative measure of centrality stability. Cases were progressively dropped from 5% to 75% of the sample across 10 sampling levels. The CS coefficient represents the largest proportion of participants that can be removed while retaining a correlation of at least 0.70 with the centrality estimates obtained from the original network with 95% probability. Following methodological guidelines, a CS coefficient of at least 0.25 is considered acceptable, with values above 0.50 indicating strong stability (Epskamp et al., 2018).

#### 2.3.4. Group Comparison

Network structures were compared between male and female participants to examine potential gender-related differences. The analysis was conducted using the R package Network Comparison Test (NCT; version 2.2.3) (van Borkulo et al., 2023). Two types of invariance tests were performed. Global tests assessed differences in overall connectivity (global strength) and network structure, assuming that both networks were identical in these aspects. Local tests evaluated differences in individual edge strength, assuming that the strength of each edge was comparable across groups.

## 3. Results

### 3.1. Descriptive Statistics and Gender Differences

Table 1 summarizes the descriptive statistics. The sample consisted of 488 participants, of whom 36.1% were male and 63.9% female, representing various regions of Oman. Significant gender differences were found for Nomophobia (t = 2.67, *p* = 0.008), FoMO (t = 2.00, *p* = 0.045), and Happiness (t = 3.90, *p* < 0.001). Specifically, females reported higher nomophobia (M = 75.80, SD = 22.40) than males (M = 70.29, SD = 20.70), and higher happiness (M = 57.30, SD = 10.28) compared to males (M = 53.11, SD = 11.80). In contrast, males showed slightly higher FoMO (M = 27.98, SD = 7.23) than females (M = 26.61, SD = 7.28). No significant gender difference was observed for mindfulness, with males scoring M = 44.30 (SD = 11.80) and females scoring M = 43.51 (SD = 11.60), t = 0.71, *p* = 0.476.

Next, Mahalanobis distance was applied to detect multivariate outliers. Using the χ^2^ critical value corresponding to *p* < 0.001, 26 cases exceeded the threshold and were removed, resulting in a final analytic sample of 462 participants in estimating network structure. This ensured that the subsequent analyses were conducted on a dataset free from extreme multivariate deviations.

### 3.2. Network Structure

**Figure 3:**
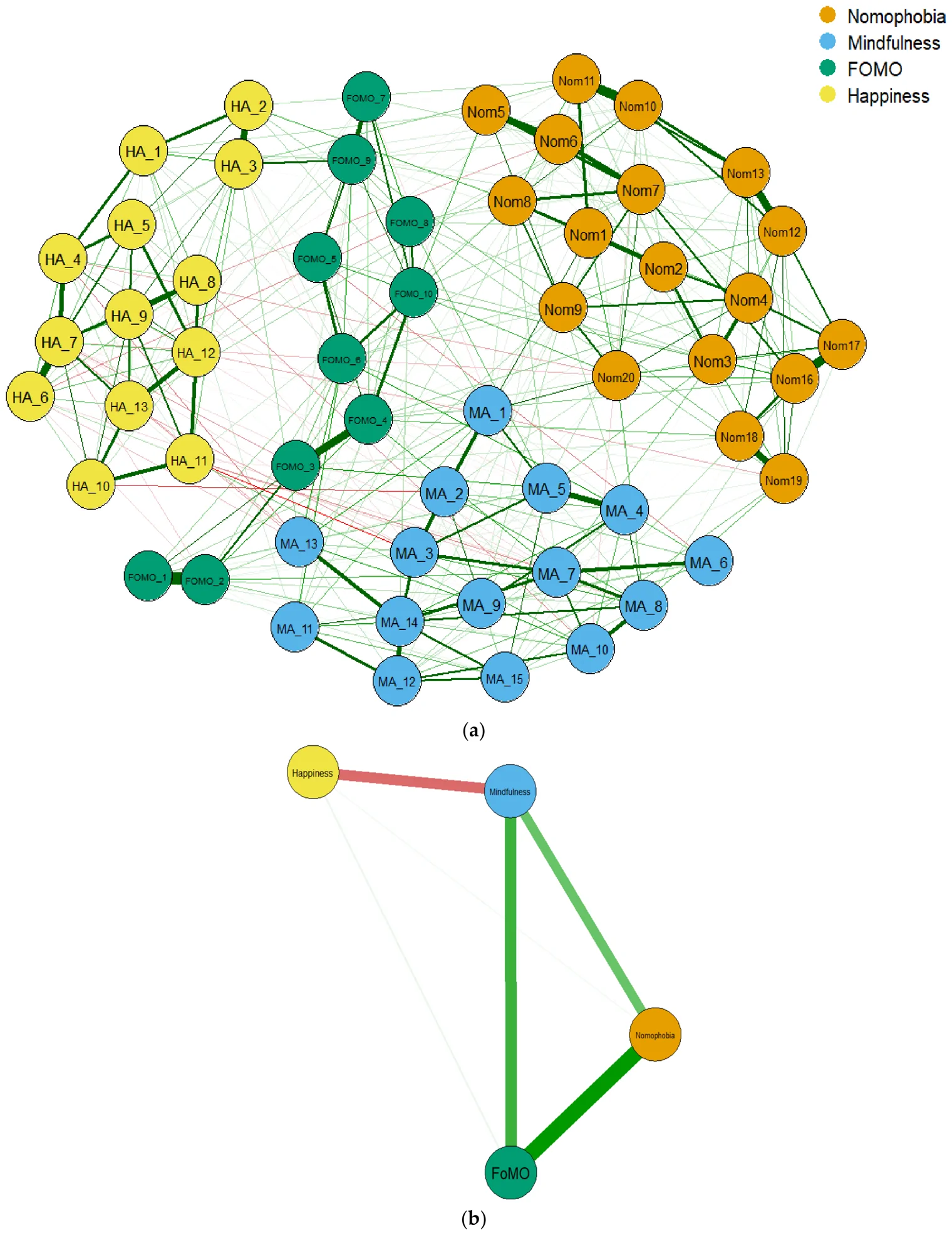
Network structure of Mindfulness, Nomophobia, FoMO, and Happiness (*n* = 462). (**a**) Item-level network structure and (**b**) Variable-level network structure. Green edges represent positive associations, while red edges represent negative associations. Orange circles represent Nomophobia items, blue circles represent Mindfulness items, green circles represent FoMO items, and yellow circles represent Happiness items.

All network analyses were conducted on the resulting analytic sample of *N* = 462. Figure 3a depicts the network structure consisting of 56 nodes (items). Using the EBIC-optimized model, 410 of the 1540 possible edges were estimated as non-zero, indicating the most substantial associations among the nodes. Overall, the network revealed strong within-community connectivity and notable cross-community links. Specifically, positive associations were detected between mindfulness, nomophobia, and FoMO nodes. Mindfulness nodes showed negative associations with Happiness.

At the community level, Figure 3b depicts the network of the four domain totals (Nomophobia, Mindfulness, FoMO, and Happiness). In this network, Edge thickness represents the strength of these associations, with thicker edges indicating stronger partial correlations. As shown in Figure 3b, the network revealed positive associations among Mindfulness, Nomophobia, and FoMO, indicating that higher mindfulness scores were related to higher levels of nomophobia and FoMO in this sample. Interestingly, happiness only showed a direct negative association with mindfulness, indicating that participants with greater mindfulness reported lower levels of happiness. Overall, Mindfulness emerged as a central bridging node, showing substantial connections to all three other constructs and highlighting its key role in the network structure.

**Figure 4:**
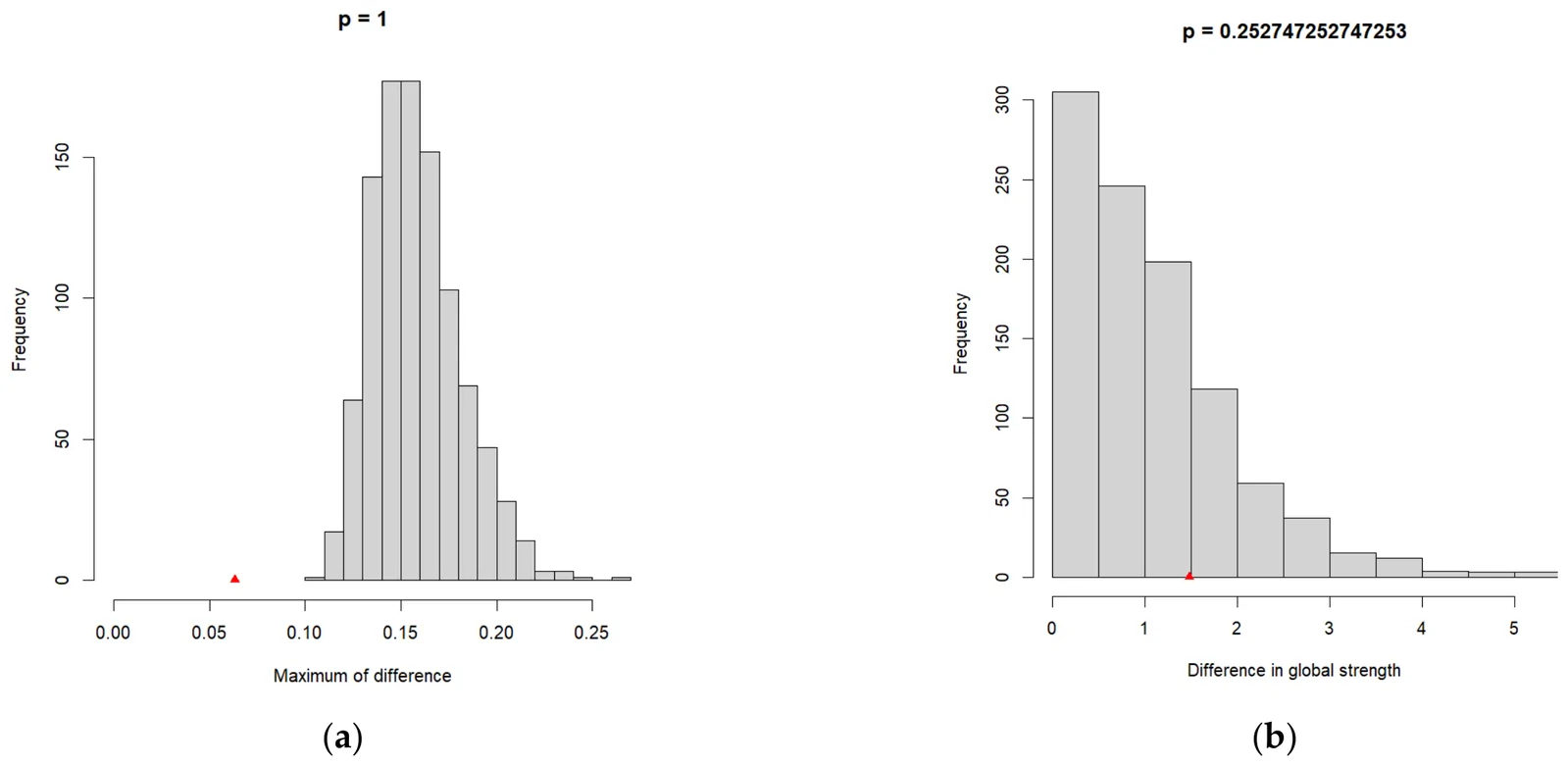
Difference in global network strength between the cleaned sample (*n* = 462) and the full sample (*n* = 488): (**a**) Network structure comparison, (**b**) Global network strength comparison. The red triangle indicates the observed difference, and the histogram represents the permutation distribution.

To assess the robustness of the network findings to the exclusion of multivariate outliers, we re-estimated the network using the full sample (*N* = 488), including the 26 participants identified by the Mahalanobis distance procedure. The full-sample network was compared with the primary network based on the cleaned sample (*N* = 462) using the Network Comparison Test (NCT). The NCT indicated no significant difference in network structure between the two samples (M = 0.063, *p* = 1.000) (see Figure 4a). Similarly, global network strength did not differ significantly between the full sample (23.708) and the cleaned sample (25.193; S = 1.485, *p* = 0.253; see Figure 4b). These findings suggest that excluding the 26 multivariate outliers did not materially affect the network structure or overall network connectivity. The full-sample network is presented in Supplementary Figure S1.

### 3.3. Centrality Measures

**Figure 5:**
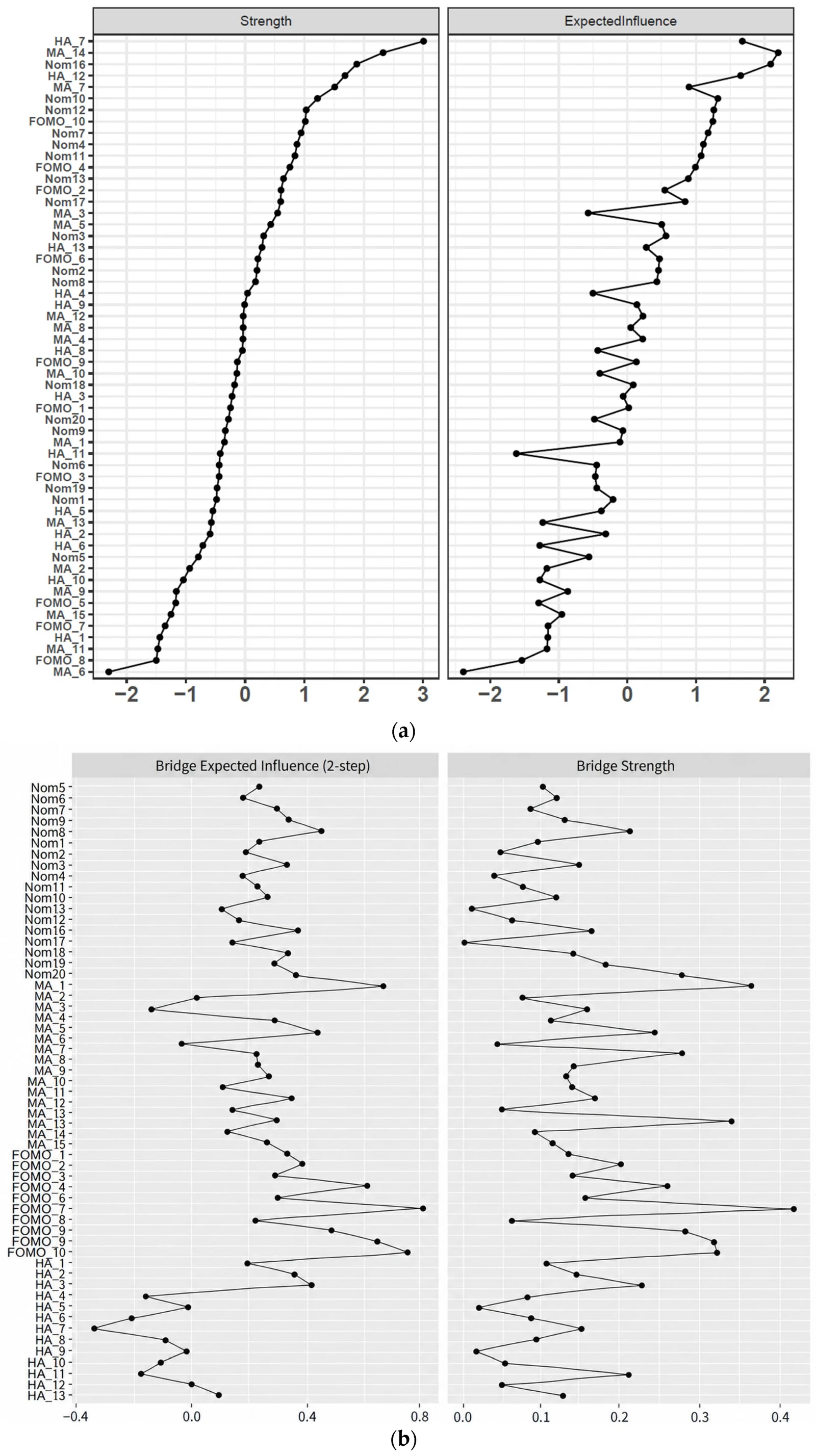
Analysis of centrality and bridge indices. (**a**) Centrality index of the network (z-scores). (**b**) Bridge centrality index of the network.

Strength and expected influence were used to determine the most central nodes in the estimated network. As shown in Figure 5 and Table 2, the analysis of Strength centrality highlighted several key nodes. HA7 (“Q15: I am very happy”) and MA14 (“I find myself doing things without paying attention”) exhibited the highest strength values, followed by Nom16 (“I would be nervous if disconnected from my online identity”), HA12 (“Q22: I often experience joy and elation”), and MA7 (“It seems I am ‘running on automatic,’ without much awareness of what I’m doing”) (See Figure 5a). These nodes play the most influential roles within the network.

Regarding expected influence, MA14 again had the highest value, followed by Nom16, HA7, HA12, and Nom10 (“If I did not have my smartphone with me, I would feel anxious because I could not instantly communicate with my family and/or friends”). These nodes have strong connections with other nodes in the network, particularly the Mindfulness item MA14 “I find myself doing things without paying attention,” which appears central in linking mindfulness-related behaviors with other constructs (See Figure 5a).

Additionally, bridge node analysis indicated that several FOMO and mindfulness items function as key connectors between communities within the network. The top bridge nodes included FOMO_6 (“Sometimes, I wonder if I spend too much time keeping up with what is going on”), MA_1 “I could be experiencing some emotion and not be conscious of it until sometime later”, FOMO_10 (“When I go on vacation, I continue to keep tabs on what my friends are doing”), FOMO_9 (“When I miss out on a planned get-together, it bothers me”), FOMO_4 (“I get anxious when I don’t know what my friends are up to”), and Nom_8 (“If I could not use my smartphone, I would be afraid of getting stranded somewhere”) (See Figure 5b). These items act as critical links connecting different psychological constructs in the network. Furthermore, the bridge expected influence at step 2 showed that FOMO_6 was the most substantial bridge node, followed by FOMO_10, MA_1, and FOMO_9 (See Figure 5b), and that these nodes are strongly connected to Nomophobia symptoms and happiness.

### 3.4. Robustness Analysis Using Ordinal Correlations

To examine the robustness of the findings to the ordinal nature of the 5-point and 6-point items, a supplementary network was estimated using cor_auto, which uses polychoric correlations for ordinal variables. The overall network structure was highly similar to that obtained using the primary EBICglasso analysis. The main centrality patterns, including strength and expected influence, were also consistent across the two approaches. Similarly, the pattern of bridge expected influence was largely unchanged. These findings indicate that the substantive conclusions were robust to the approach used to account for the ordinal response structure. Detailed results are provided in Supplementary Figures S2–S4.

### 3.5. Accuracy and Stability Estimation

**Figure 6:**
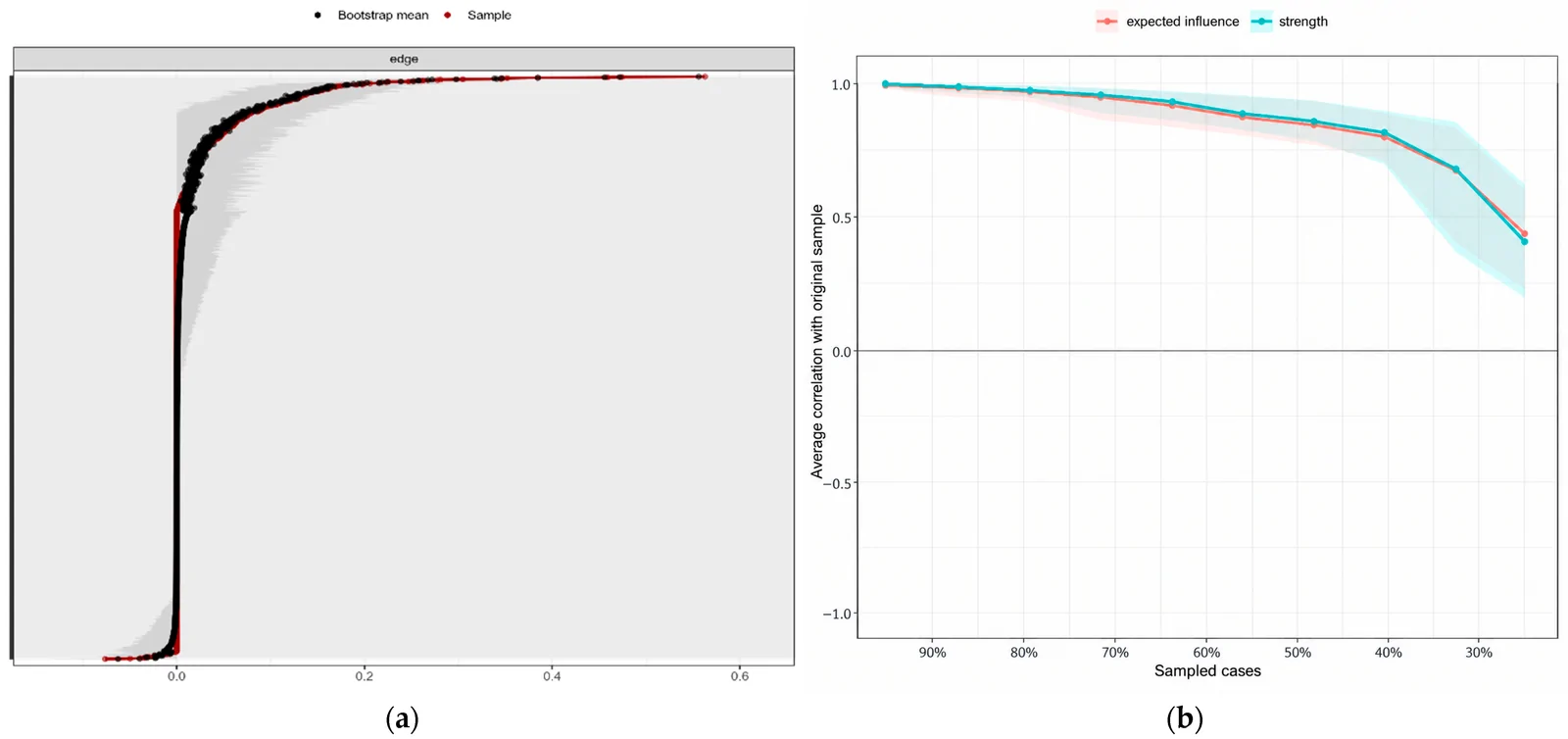
Network robustness and accuracy. (**a**) Robustness. (**b**) Accuracy.

The bootstrapped accuracy assessment showed that the confidence intervals of the edge weights were relatively narrow (Figure 6a), indicating good precision in the estimated connections. As shown in Figure 6b, the stability analysis demonstrated that the correlation stability coefficients were 0.595 for strength and 0.517 for expected influence. These values exceed the recommended minimum threshold of 0.50, suggesting that both centrality indices remained adequately stable even when a substantial proportion of cases was removed. Overall, the results support the accuracy and stability estimation of the network estimates.

### 3.6. Network Gender Comparison Test

**Figure 7:**
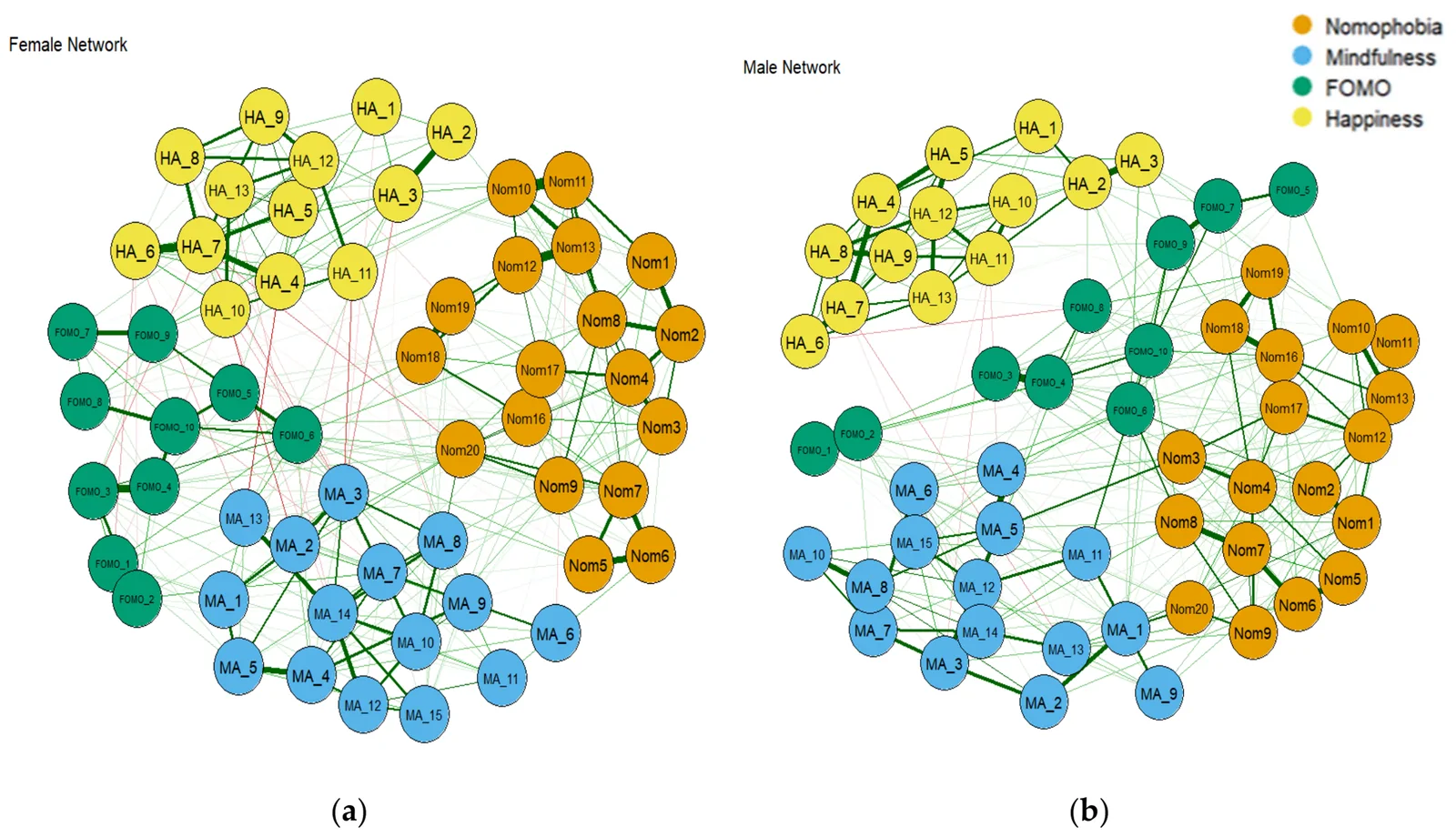
Network structure for female and male. (**a**) Female network. (**b**) Male network. Green edges represent positive associations, while red edges represent negative associations. Orange circles represent nomophobia items, blue circles represent mindful attention items, green circles represent FoMO items, and yellow circles represent happiness items.

The NCT indicated no significant difference in the overall network structure between females and males (M = 0.22, *p* = 0.40; see Figure 7). Similarly, the comparison of global strength showed that the two networks did not differ significantly (S = 3.08, *p* = 0.58). These findings indicate that no statistically significant gender differences were detected in the network structure or global strength of Nomophobia, FoMO, happiness, and mindfulness.

## 4. Discussion

The present study employed network analysis to examine item-level associations among nomophobia, FoMO, mindfulness, and personal happiness in Omani college students. Contrary to expectations derived from Western mindfulness literature, mindful attention (as measured by the MAAS) was positively associated with technology-related constructs (nomophobia and FoMO) and negatively associated with happiness. Rather than functioning as a protective psychological buffer, mindfulness showed a central position linking digital-related constructs and happiness within the estimated network. Bridge node analysis further revealed that FoMO items—particularly those reflecting concerns about staying socially updated (FOMO_6) and maintaining social contact during absences (FOMO_10)—served as key connectors across communities, highlighting prominent associations across the network.

### 4.1. The Complex Relationship Between Mindful Attention and Happiness

Mindful attention functioned as a key bridging node, displaying strong links to all three other constructs (nomophobia, FoMO, and happiness), highlighting its key role in the network structure. Mindfulness showed positive linkages with both nomophobia and FoMO nodes, while its connections with happiness nodes were negative. Furthermore, the present pattern of positive associations between mindful attention and both nomophobia and FoMO diverges from the assumption that dispositional mindfulness is uniformly protective, suggesting instead that its relationship with well-being may be context-dependent (Baer et al., 2021). This is consistent with the broader observation that dispositional mindful attention is not uniformly associated with happiness or well-being across individuals. Knowing that MAAS is a measure of present awareness but not other variables of mindfulness (i.e., acceptance, nonjudgement, compassion, and decentering), it may be understandable that mindful attention is context specific. Social comparison concerns, disconnection anxiety, and internal discomfort may coexist with mindful attention, although the present cross-sectional design does not allow for conclusions about the direction of these associations.

### 4.2. Mindfulness and Happiness Can Be Negatively Correlated

Nodes such as HA7 (“Q15: I am very happy”) and MA14 (“I find myself doing things without paying attention”) exhibited the highest strength values. In light of the classical theory of mindfulness (Kabat-Zinn, 2015), individuals permit themselves to experience whatever arises when confronted with a threatening event without suppression of other responses, such as distraction. The same interesting findings for Nom16 (“I would be nervous if disconnected from my online identity”), HA12 (“Q22: I often experience joy and elation”), and MA7 (“It seems I am ‘running on automatic,’ without much awareness of what I’m doing”) showed notable associations within the network. The number of hours on SNS may not be a direct predictor of happiness, especially if SNS is used for social connection (Arampatzi et al., 2018). Simply observing thoughts, feelings, and sensations repeatedly is unlikely to automatically produce cognitive insights such as “thoughts are not facts” or “my feeling of being a bad person is merely a thought” (Rapgay & Bystrisky, 2009). Given that MAAS measures present moment attention, the negative association between mindful attention and happiness may reflect the specific dimension of mindfulness captured by the MAAS. In other words, subjective happiness may not necessarily be associated with present-moment attention alone, particularly when other components of mindfulness are not assessed.

### 4.3. Social Media as a Source of Temporal Happiness

Previous studies conducted within the Omani culture reported that adolescents employed more escape coping as problem-focused avoidance. The theme of avoidance, whether physically immersing oneself in different activities or mentally avoiding the thought of the painful experience, was a coping strategy reported by students (Al-Bahrani et al., 2013). Likewise, the use of suppression and avoidant coping can be detrimental to adaptive functioning for those in collectivistic cultures, even though each strategy aligns with their cultural conventions. Fear of happiness was strongly and positively correlated with denial. The strategy of denial, venting, and self-blame could be used to manage and suppress positive emotions by individuals who perceive intense positive emotions as threatening or overwhelming (Gandhi, 2024; Hirano & Ishii, 2024). Indeed, humans are biologically predisposed to automatically evaluate environmental information, with initial assessments of potential stressors occurring at a subcortical level before conscious awareness or emotion (Britton et al., 2021). These findings provide possible contextual explanations for the observed associations, but they do not establish causal relationships.

### 4.4. Mindful Attention Shows Positive Associations with Nomophobia and FoMO

Positive relationships were observed among the mindfulness, nomophobia, and FoMO nodes. In contrast, Arpaci et al. (2017) found a significant direct effect of mindfulness on nomophobia, specifically for women. Women with higher levels of mindfulness were less likely to report nomophobia. Other studies indicated a small negative association between trait mindfulness and nomophobia, suggesting that individuals with higher levels of trait mindfulness tended to report lower levels of nomophobia (Fendel et al., 2025). The positive associations observed in the present study therefore differ from some previous findings and may reflect differences in measurement, samples, or cultural context. In contexts characterized by constant connectivity, greater mindful awareness may be associated with greater awareness of experiences related to nomophobia and FoMO.

When individuals observe the contents of their consciousness, they are no longer fully immersed in or fused with those experiences. To notice an experience implies that one is more than the experience itself, whether it involves pain, depression, or fear. Reperceiving enables individuals to step back from thoughts, emotions, and bodily sensations as they arise, allowing them to relate to these experiences without being defined or controlled by them (Shapiro et al., 2006). In that sense, loneliness is closely tied to fundamental social needs, particularly the need for relatedness and belonging. When individuals engage in social comparison, they seek similarity or social validation, and thus unmet social needs may be associated with feelings of loneliness. This loneliness, in turn, has also been associated with FoMO, as individuals strive to enhance their sense of connection (Piko et al., 2025). These findings provide a possible context for understanding the observed association between mindful attention and FoMO, although the present findings do not establish a causal relationship. Wells’ Self-Regulatory Executive Function (S-REF) model (Wells & Matthews, 1994) suggests that an external attentional focus might help people with anxiety disorders, rather than an internal focus as one would use in mindfulness training. In Wells’ S-REF terms, mindfulness shifts metacognitions from viewing thoughts as personal and threatening to seeing them as impersonal and transient, adopting a decentral perspective (Shapiro et al., 2006). Therefore, Wells’ S-REF model describes a cognitive attentional syndrome marked by excessive self-focused attention, threat monitoring, rumination, and dysfunctional beliefs, which are linked to disorders like anxiety. The model suggests that focusing internally on anxiety-related sensations may be related to fear and negative thinking. In contrast to mindfulness-based approaches (IAA model), the S-REF intervention emphasizes external attentional focus monitoring and switching attention outwardly, often using auditory tasks. Comparing the effectiveness of internal (mindfulness) versus external (S-REF) attentional strategies in treating anxiety could provide valuable empirical insights (Shapiro et al., 2006). However, such causal or comparative questions require longitudinal or experimental research.

### 4.5. Theoretical Contributions

Mindfulness was found to be negatively associated with happiness. Although the integration of mindfulness into Western psychology has generated considerable enthusiasm and encouraging preliminary findings, the results of the present study tentatively raise questions regarding the conceptualization of mindfulness and its relationship to positive emotions, such as happiness, within some collectivist cultures; however, as noted in the Limitations, cultural explanations were not directly measured in this study and should be treated as hypotheses for future research rather than conclusions. Western operationalizations of mindfulness have demonstrated validity through their associations with Western psychological constructs. However, mindfulness may not readily generalize to some other cultural contexts. For example, the distinctions between observing, describing, acting with awareness, and accepting without judgment may not be applicable, and that a more flexible or integrated conceptualization of mindfulness and related constructs might be more appropriate. Furthermore, mindfulness practices and techniques have been incorporated into structured clinical interventions. To address growing demand in therapeutic settings, the conceptualization of mindfulness has shifted from its classical roots to a more therapy-oriented perspective, with the addition of the component of acceptance. This evolution highlights the need for a comprehensive assessment tool suitable for various populations that captures the essential dimensions of mindfulness (Christopher et al., 2009; Chan et al., 2015). Prior theoretical work similarly suggests that mindfulness is not universally beneficial and that its relationship with well-being may depend on individual and contextual factors, consistent with the present pattern of associations (Baer et al., 2021). As this study assessed dispositional mindful attention rather than meditation practice or intervention, findings from meditation-related adverse-effects research are not directly comparable and were not used to interpret the present results.

SNSs have become a place for social connectedness among young adults (Manago & Vaughn, 2015). Thus, the assumption that moderate or high levels of smartphone and social media use are not necessarily associated with decreased happiness or deteriorated mental health (Arampatzi et al., 2018). In fact, for many individuals, SNS is a source of gratification, social connection, and happiness, despite some concerns regarding life satisfaction and self-worth (Manago & Vaughn, 2015). Mindfulness may not uniformly operate as a psychological buffer in digital contexts. In the present network, mindful attention was positively associated with FoMO and nomophobia and negatively associated with happiness, suggesting a complex pattern of associations rather than a uniformly protective pattern. These findings should not be interpreted as evidence that mindful attention amplifies distress or causes lower happiness. Given that the MAAS primarily measures attentional awareness, these findings should be interpreted specifically in terms of mindful attention rather than mindfulness as a broader construct. The findings highlight the value of network approaches for understanding the complex associations among mindful attention, technology-related concerns, and digital well-being.

### 4.6. Limitations

The current study has several limitations. The first limitation is the influence of extraneous factors that were not measured by the current study such as financial stress, academic stress, psychological capital, living away from family, and familial issues. Another limitation relates to the timing of data collection. Data was collected over a period of a few weeks and may sometimes be conflated with other demands such as exams and assignment due dates, which may have affected perception of each of the variables. Third, mindfulness was measured by MAAS, which is a measure of present moment attention and does not include all facets of mindfulness (i.e., non-judgement, acceptance, emotion regulation skills). Thus, our focus was on one main aspect of mindfulness. Network structure may look different if other facets were measured. Fourth, understanding of mindfulness and happiness may be culturally unique, despite careful translation and back translation work. The idea of happiness is generally associated with satisfaction while mindfulness is usually seen from a religious lens. Thus, findings should be handled with care in other settings or cultures. Fifth, although the NCT did not identify significant gender differences in network structure or global strength, the unequal group sizes may have limited statistical power, and measurement invariance was not established before the gender comparison. Therefore, these findings should not be interpreted as definitive evidence of network equivalence across gender. Future studies should use adequately powered and balanced samples and establish measurement invariance before conducting gender-based network comparisons. Finally, given the cross-sectional, self-report design, network associations and centrality and bridge indices should not be interpreted causally. Cultural explanations were not directly measured and should therefore be considered tentative interpretations.

## 5. Conclusions

This study is the first to examine network-level associations among nomophobia, FoMO, mindfulness, and happiness in an Arab college student population. Network analysis revealed that mindful attention, as operationalized by the MAAS, was positively associated with Nomophobia, and FoMO and negatively associated with happiness nodes, suggesting that the relationship between mindfulness and well-being may be more complex than a uniformly protective association. These findings highlight the importance of considering acceptance and emotion-regulation components when interpreting present-moment attention (Britton, 2019; Baer et al., 2021). Key bridge nodes—predominantly FoMO items—showed important cross-community associations that may inform future research on digital well-being. No statistically significant gender differences in network structure or global strength were detected in this sample. Future longitudinal and experimental research should examine whether the acceptance and emotion-regulation facets of mindfulness, which were not captured by the MAAS, may help explain the observed associations among mindful attention, nomophobia, and FoMO, and whether these associations vary across cultural contexts or following mindfulness-based interventions adapted to collectivistic cultural values.

## Figures and Tables

**Table 1:** Sociodemographic characteristics.

Variable	Category	*n*	(%)
Gender	Male	176	36.1
	Female	312	63.9
College	Education	327	67.0
	Nursing	106	21.7
	Arts & Social Sciences	44	9.0
	Other	11	2.2
Year of Study	Preparatory	6	1.2
	First	94	19.3
	Second	116	23.8
	Third	78	16.0
	Fourth	58	11.9
	Fifth and above	136	27.9
Cumulative GPA	<2.00	27	5.5
	2.00–2.29	45	9.2
	2.30–2.74	108	22.1
	2.75–3.29	184	37.7
	3.30–3.74	94	19.3
	3.75–4.00	30	6.1
Monthly Family Income (OMR)	≤249	28	5.7
	250–499	58	11.9
	500–749	82	16.8
	750–999	84	17.2
	1000–1249	100	20.5
	≥1250	136	27.9
Birth Order	First	107	21.9
	Middle	307	62.9
	Last	60	12.3
	Only child	12	2.5
	Other	2	0.4

Note: The category ‘other colleges’ includes Agricultural & Marine Sciences, Medicine & Health Sciences, and Science.

**Table 2:** Item-level descriptive statistics and centrality indices for all network nodes (*N* = 462).

Node	Item Content	Abbreviation	Mean	SD	ST	EI	Z_ST	Z_EI
	Nomophobia							
Nom1	I would feel uncomfortable without constant access to information through my smartphone	no access	5.51	1.69	0.80	0.80	−0.48	−0.21
Nom2	I would be annoyed if I could not look information up on my smartphone when I wanted to do so	no_lookup	4.93	1.73	0.94	0.94	0.20	0.45
Nom3	Being unable to get the news (e.g., happenings, weather, etc.) on my smartphone would make me nervous	no_news	3.44	1.88	0.96	0.96	0.31	0.56
Nom4	I would be annoyed if I could not use my smartphone and/or its capabilities when I wanted to do so	no_use	4.29	1.76	1.07	1.07	0.87	1.11
Nom5	Running out of battery in my smartphone would scare me	no_battery	3.50	1.82	0.74	0.73	−0.79	−0.56
Nom6	If I were to run out of credits or hit my monthly data limit, I would panic	no_data	3.57	1.97	0.81	0.76	−0.44	−0.45
Nom7	If I did not have a data signal or could not connect to Wi-Fi, then I would constantly check to see if I had a signal or could find a Wi-Fi network	no_signal	3.97	1.89	1.09	1.09	0.94	1.18
Nom8	If I could not use my smartphone, I would be afraid of getting stranded somewhere.	stranded	4.72	2.03	0.93	0.93	0.17	0.43
Nom9	9. If I could not check my smartphone for a while, I would feel a desire to check it.	desire_check	4.56	1.78	0.83	0.83	−0.33	−0.07
	If I did not have my smartphone with me,							
Nom10	I would feel anxious because I could not instantly communicate with my family and/or friends.	no_communicate	4.84	1.75	1.14	1.12	1.22	1.32
Nom11	I would be worried because my family and/or friends could not reach me	unreachable	4.88	1.76	1.07	1.07	0.84	1.08
Nom12	I would feel nervous because I would not be able to receive text messages and calls	no_msgs_calls	3.99	1.82	1.10	1.10	1.03	1.26
Nom13	I would be anxious because I could not keep in touch with my family and/or friends	no_contact	4.09	1.83	1.03	1.03	0.65	0.89
Nom16	I would be nervous because I would be disconnected from my online identity	no_online_id	3.40	1.92	1.27	1.27	1.88	2.09
Nom17	I would be uncomfortable because I could not stay up to date with social media and online networks	no_social_update	3.71	1.85	1.02	1.02	0.60	0.84
Nom18	I would feel awkward because I could not check my notifications for updates from my connections and online networks	no_notifications	3.06	1.80	0.86	0.86	−0.18	0.09
Nom19	I would feel anxious because I could not check my email messages	no_email	3.02	1.82	0.81	0.76	−0.47	−0.45
Nom20	I would feel weird because I would not know what to do	unsure	4.33	1.97	0.84	0.75	−0.28	−0.48
	Mindfulness							
MA_1	I could be experiencing some emotion and not be conscious of it until sometime later.	unaware_emotion	3.60	1.29	0.83	0.82	−0.35	−0.11
MA_2	I break or spill things because of carelessness, not paying attention, or thinking of something else.	careless	3.62	1.44	0.71	0.61	−0.94	−1.17
MA_3	I find it difficult to stay focused on what’s happening in the present.	hard_focus	2.83	1.32	1.01	0.73	0.55	−0.57
MA_4	I tend to walk quickly to get where I’m going without paying attention to what I experience along the way.	fast_walk	3.42	1.64	0.89	0.89	−0.04	0.22
MA_5	I tend not to notice feelings of physical tension or discomfort until they really grab my attention.	ignore_tension	3.08	1.34	0.98	0.95	0.43	0.50
MA_6	I forget a person’s name almost as soon as I’ve been told it for the first time.	forget_name	3.15	1.58	0.44	0.36	−2.30	−2.39
MA_7	It seems I am “running on automatic,” without much awareness of what I’m doing.	automatic	2.68	1.38	1.20	1.03	1.51	0.90
MA_8	I rush through activities without being really attentive to them.	rush_activities	2.79	1.41	0.89	0.86	−0.03	0.05
MA_9	I get so focused on the goal I want to achieve that I lose touch with what I’m doing right now to get there.	goal_focus	3.59	1.36	0.67	0.67	−1.16	−0.87
MA_10	I do jobs or tasks automatically, without being aware of what I’m doing.	auto_tasks	2.66	1.27	0.87	0.77	−0.14	−0.40
MA_11	I find myself listening to someone with one ear, doing something else at the same time.	partial_listen	3.15	1.47	0.61	0.61	−1.47	−1.17
MA_12	I drive places on ‘automatic pilot’ and then wonder why I went there.	auto_drive	2.78	1.52	0.89	0.89	−0.03	0.23
MA_13	I find myself preoccupied with the future or the past.	future_past	3.89	1.58	0.79	0.60	−0.57	−1.23
MA_14	I find myself doing things without paying attention.	no_attention	2.95	1.37	1.36	1.29	2.33	2.20
MA_15	I snack without being aware that I’m eating.	auto_snack	2.45	1.55	0.65	0.65	−1.25	−0.96
	Fear of Missing Out							
FOMO_1	I fear others have more rewarding experiences than me	fear_others	2.47	1.24	0.85	0.85	−0.25	0.02
FOMO_2	I fear my friends have more rewarding experiences than me.	fear_friends	2.44	1.23	1.02	0.96	0.60	0.55
FOMO_3	I get worried when I find out my friends are having fun without me.	worry_miss	2.46	1.31	0.81	0.75	−0.44	−0.47
FOMO_4	I get anxious when I don’t know what my friends are up to.	anxious_unknown	2.05	1.15	1.05	1.05	0.75	0.99
FOMO_5	It is important that I understand my friends ‘‘in jokes’’.	know_jokes	3.24	1.29	0.67	0.58	−1.17	−1.29
FOMO_6	Sometimes, I wonder if I spend too much time keeping up with what is going on.	too_much_time	3.06	1.22	0.94	0.94	0.21	0.47
FOMO_7	It bothers me when I miss an opportunity to meet up with friends.	miss_opportunity	2.94	1.24	0.63	0.61	−1.35	−1.16
FOMO_8	When I have a good time, it is important for me to share the details online (e.g., updating status).	share_online	2.23	1.21	0.60	0.53	−1.50	−1.54
FOMO_9	When I miss out on a planned get-together it bothers me.	miss_event	3.51	1.22	0.87	0.87	−0.13	0.13
FOMO_10	When I go on vacation, I continue to keep tabs on what my friends are doing.	vacation_check	2.70	1.25	1.10	1.10	1.01	1.25
	Happiness							
HA_1	Q3: I feel that life is very rewarding	life_rewarding	3.95	1.43	0.62	0.61	−1.44	−1.16
HA_2	Q4: I have very warm feelings towards almost everyone	warm_feelings	4.09	1.28	0.78	0.78	−0.59	−0.31
HA_3	Q8: I am always committed and involved	committed	3.92	1.26	0.86	0.83	−0.22	−0.06
HA_4	Q9: life is good	life_good	4.58	1.46	0.91	0.74	0.04	−0.50
HA_5	Q11: I laugh a lot	laugh	4.41	1.38	0.79	0.77	−0.54	−0.38
HA_6	Q12: I am well satisfied about everything in my life	satisfied	4.63	1.28	0.76	0.59	−0.71	−1.28
HA_7	Q15: I am very happy	very_happy	4.30	1.33	1.49	1.19	3.01	1.68
HA_8	Q16: I find beauty in some things	find_beauty	4.81	1.16	0.89	0.76	−0.05	−0.43
HA_9	Q17: I always have a cheerful effect on others	cheerful_effect	4.40	1.26	0.90	0.88	−0.01	0.14
HA_10	Q18: I find time for everything	time_for_all	3.55	1.33	0.69	0.59	−1.04	−1.27
HA_11	Q21: I feel fully mentally alert	mentally_alert	4.48	1.23	0.82	0.52	−0.42	−1.62
HA_12	Q22: I often experience joy and elation	joy_elation	4.46	1.20	1.23	1.18	1.68	1.65
HA_13	Q25: I feel I have a great deal of energy	energy	4.31	1.30	0.96	0.90	0.28	0.28

Note. ST = raw values of Strength; EI = raw values of Expected Influence; Z_ST = z-scores of Strengths; Z_EI = z-scores of Expected Influence.

## Data Availability

Data and code supporting the findings of this study are available from the corresponding author upon reasonable request.

## Supplementary Materials

The following supporting information can be downloaded at: https://www.mdpi.com/article/10.3390/bs16091613/s1, Figure S1. Item-level network structure of Mindfulness, Nomophobia, FoMO, and Happiness based (*n* = 488). Figure S2. Item-level network structure of Mindfulness, Nomophobia, FoMO, and Happiness based on ordinal correlations (*n* = 462). Figure S3. Centrality index of the network(z-scores) based on ordinal correlations (*n* = 462). Figure S4. Bridge centrality indices of the network based on ordinal correlations (*n* = 462).
