# Supplementary material for: Network Structure of Nomophobia, Fear of Missing Out, Mindful Attention, and Happiness Among University Students
Source: Behav Sci (Basel). 2026 Sep 9;16(9):1613. doi: 10.3390/bs16091613 (PMC13603627; doi:10.3390/bs16091613)
Supplement: Supplementary file 1 [file behavsci-16-01613-s001.zip › behavsci-4437256-supplementary.pdf]

Supplemental Materials for

Network Structure of Nomophobia, Fear of Missing Out, Mindfulness, and Happiness in Omani College Students

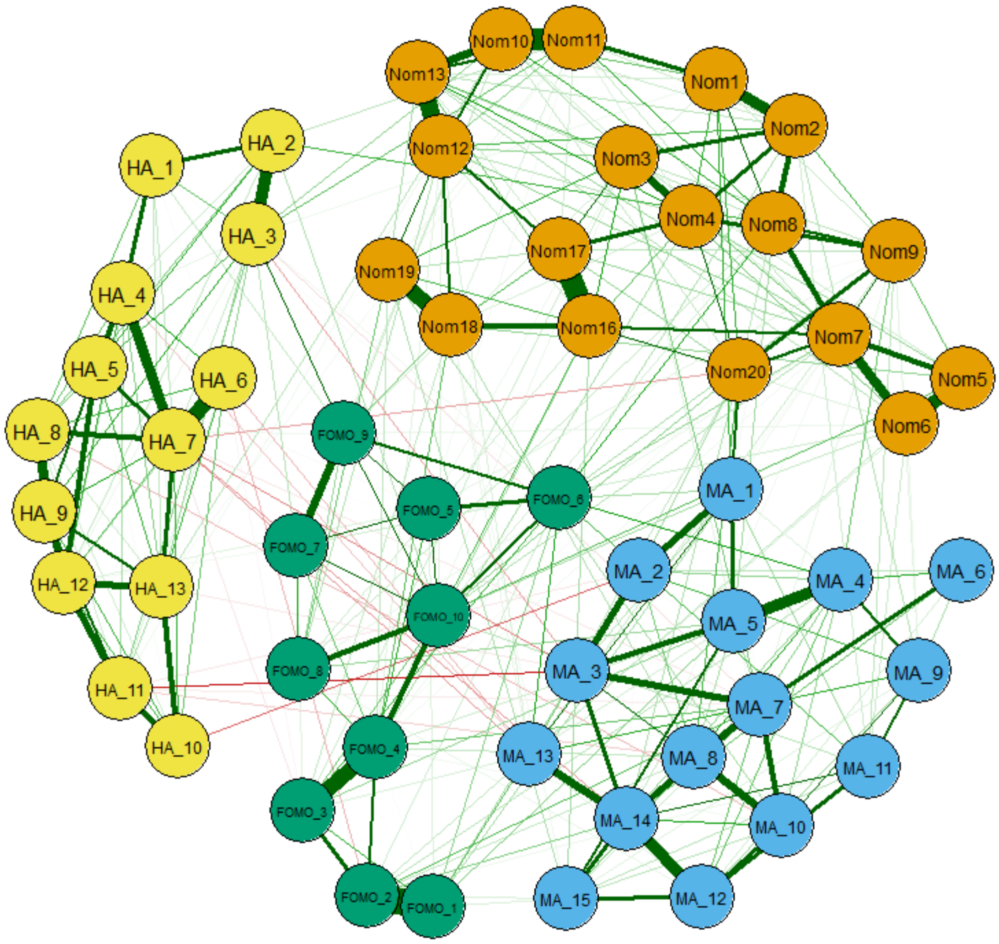

Figure S1. Item-level network structure of Mindfulness, Nomophobia, FoMO, and Happiness based (n= 488)

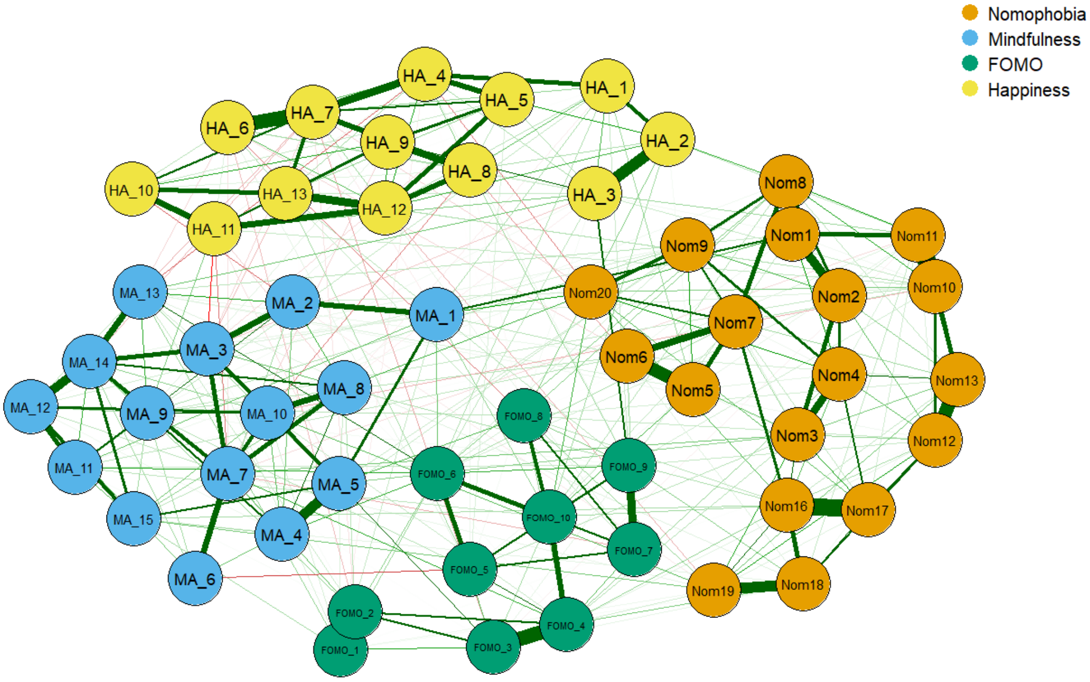

Figure S2. Item-level network structure of Mindfulness, Nomophobia, FoMO, and Happiness based on ordinal correlations (n = 462).

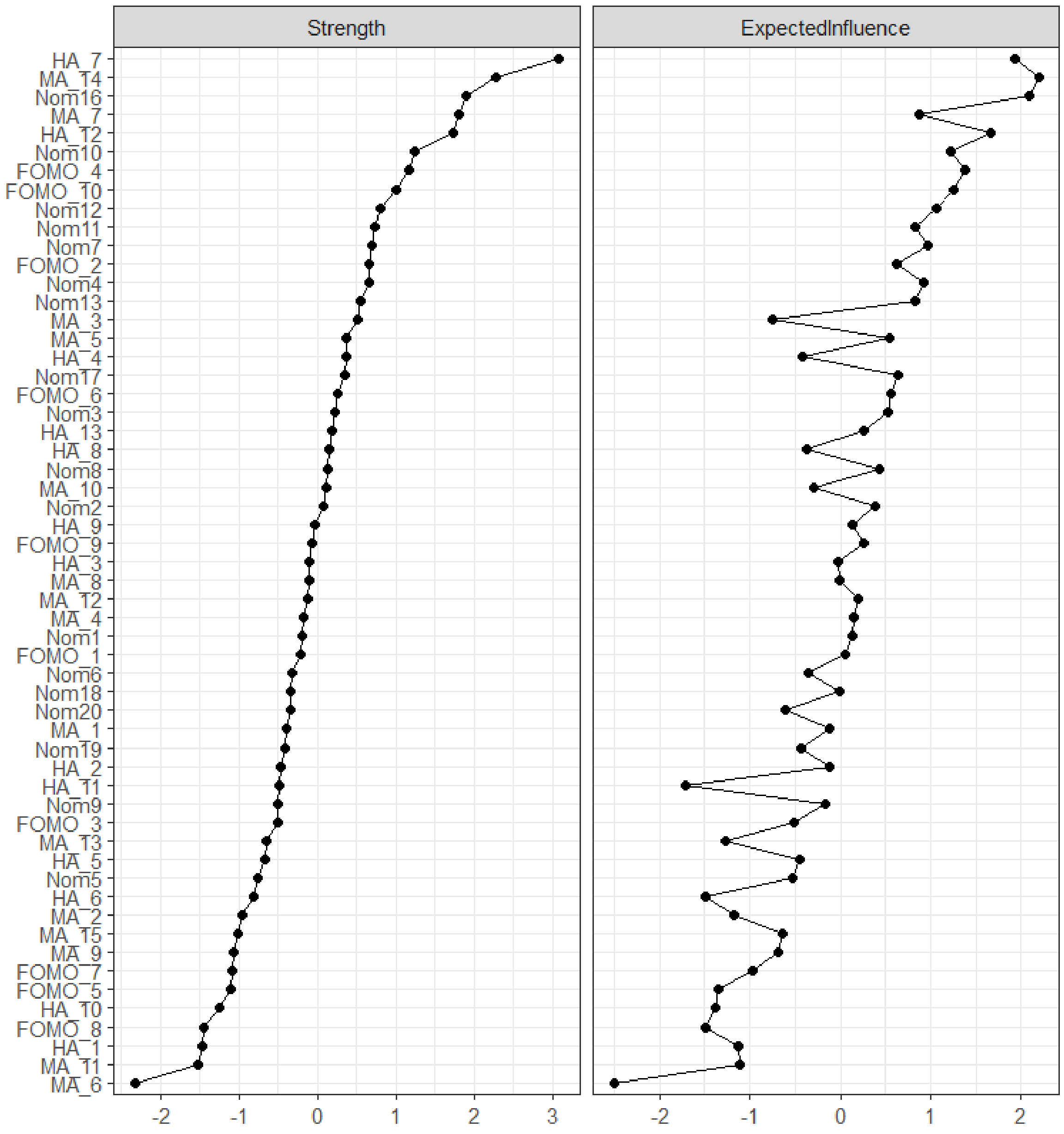

Figure S3. Centrality index of the network(z-scores) based on ordinal correlations (n = 462).

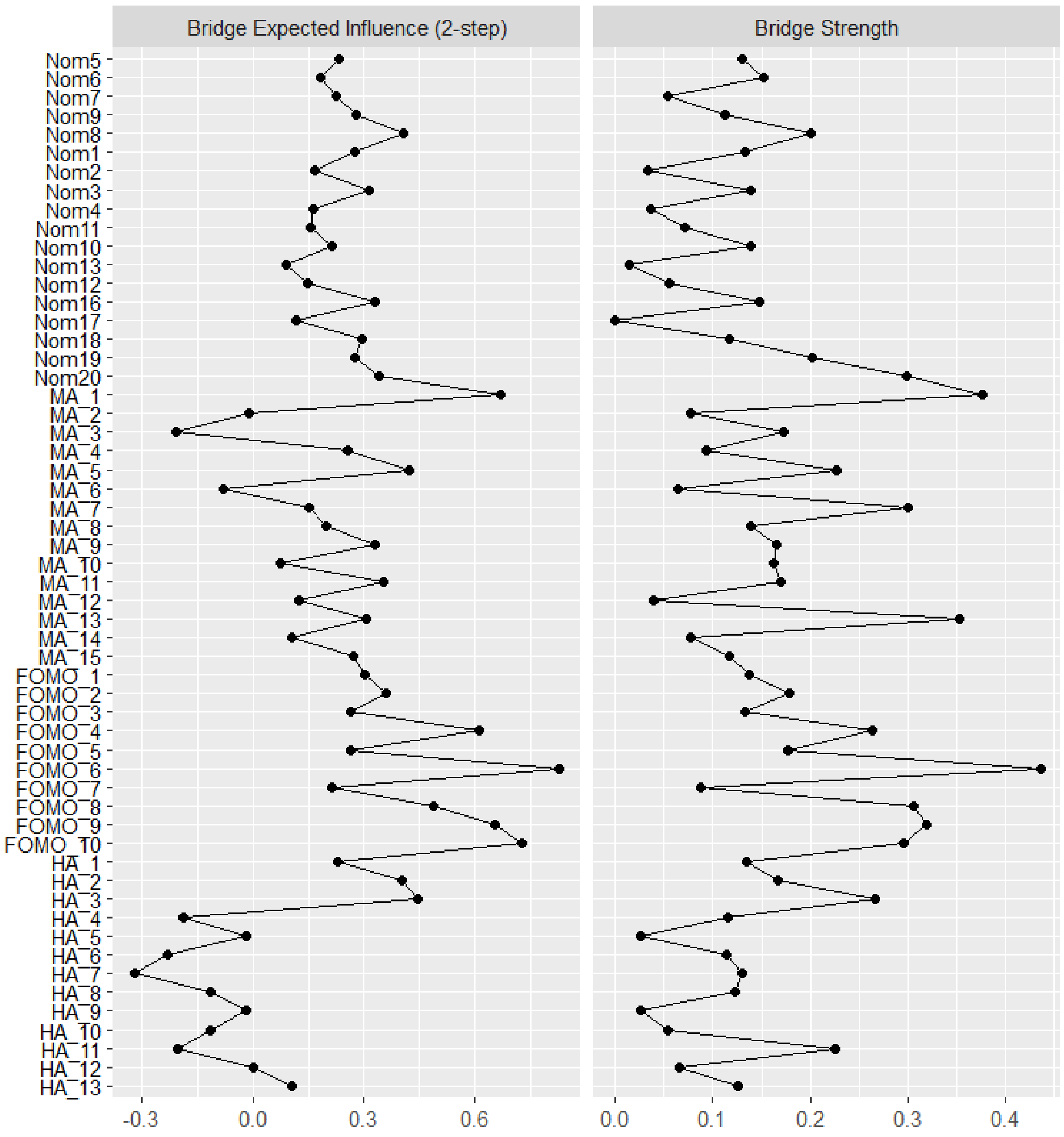

Figure S4. Bridge centrality indices of the network based on ordinal correlations (n = 462).
